# Colorimetry characteristics and color clustering of natural gem-quality spinel from Myanmar (Burma)

**DOI:** 10.1371/journal.pone.0312054

**Published:** 2025-08-14

**Authors:** Jun Tang, Wenfang Zhu, Kaichao Liu, Ying Guo

**Affiliations:** 1 School of Gemmology, China University of Geosciences (Beijing), Beijing, China; 2 Asian Institute of Gemological Sciences (China), Shenzhen, Guangdong, China; 3 Shenzhen Laboratory, National Gemstone Testing Center, Shenzhen, Guangdong, China; National Research Centre, EGYPT

## Abstract

Color is the most critical factor in determining the value of gem-quality spinel. This paper examines the color mechanism and the colorimetric characteristics of spinel crystals under D65 and A light sources against nine neutral backgrounds. It also explores color clustering for over 400 spinel crystals in yellow, red, purple, and blue hues. Various analytical techniques were employed, including optical absorption spectroscopy, Energy-Dispersive X-ray Fluorescence analysis, a benchtop sphere spectrophotometer, the Munsell neutral value gray scale chart, and a standard illumination box. The study reveals distinct optical absorption peaks corresponding to different spinel colors, with these peaks being assigned to specific mechanisms based on previous research. Color analysis demonstrates that the chroma and hue of orange spinel crystals are primarily influenced by the red tone under both daylight and incandescent light. Additionally, red, and yellow tones significantly enhance the color of red spinel. For purple spinel, chroma shows a strong correlation with the a^*^ value under both D65 and A light sources, while hue is easily influenced by the blue tone. In blue spinel, chroma is controlled by the green tone, and hue is affected by the blue tone. Lightness and chroma of all spinel colors increase significantly with the luminance factor of the Munsell neutral background, following a power function relationship with varying rates. However, the hue angle remains relatively unchanged, as gray backgrounds have minimal effect on hue. The colors of spinel crystals can be effectively predicted using calculated equations based on the luminance factor of the background under different light sources. The K-means clustering method is particularly effective for categorizing each spinel color into three distinct groups, which is crucial for developing a reliable color grading system for spinel.

## Introduction

Color is a visual perception based on the electromagnetic spectrum. There are two primary concepts related to color: body color and color appearance. Body color results from the reflection and selective absorption of certain wavelengths of light by the colored substance [1]. Color appearance is more about how humans perceive color under various conditions such as light source, background, and illumination level [2]. Understanding color is vital, as it significantly influences material value and is a fundamental physical property often used in material identification [3].

Spinel oxides, with the general formula AB_2_O_4_ (where A atoms are tetrahedrally coordinated and B atoms are octahedrally coordinated), constitute a large group of minerals. These minerals are frequently used as gemstones or ceramic pigments due to their diverse and intense colors [4]. High-quality natural spinels are found worldwide, including in Myanmar (both the Mogok Valley and the Namya area) [5–7], Madagascar, Vietnam (Luc Yen District) [8–9], Afghanistan [9], Sri Lanka, Tajikistan, and Tanzania (Mahenge, Uluguru Mountains, and Tunduru) [7,10]. The spinel from Myanmar is particularly renowned for its vivid color, high quality, and historical significance.

The color of gem-quality spinel is the most critical factor determining its value. Spinels exhibit a broad spectrum of colors due to the presence of transition metal elements [11]. Common spinel colors include orange, red, purple, blue, and pink, while colorless, green, and yellow spinels are less frequently encountered. The body color of spinel is influenced by the combined effects of multiple transition metals, such as Cr^3+^, Fe^2+^, Fe^3+^, Mn^2+^, Co^2+^, Cu^2+^, and V^3+^. There are fifteen color mechanisms in nature have been identified by Nassau [12], involving fundamental processes like physical optics, geometrical considerations, molecular orbitals, energy bands, vibrations, simple excitations, and ligand-field effects. Thirteen of those occur in the area of mineralogy, for example, vibrations and rotations (ice), transition metal compounds (turquoise), transition metal impurities (ruby, emerald…), organic compounds (amber), charge transfer (blue sapphire), metals (gold), pure semiconductors (diamond), doped or activated semiconductors (yellow diamond), color center (amethyst), dispersive refraction and polarization (“fire” in gemstones), scattering (moonstone), interference (tarnish of minerals), and diffraction (opal). Except for the 13 causes of color mentioned above, there are more color mechanisms were studied in recent years. For example, a complex color mechanism involves structural relaxation around Cr^3+^, which affects light absorption in spinel [13]. In spinel, color mechanisms generally include electronic transitions within the d orbitals of transition metal cations and charge transfer between oxygen and ligands [14].

It is important to note that the perceived color appearance of a transparent mineral is a composite effect influenced by its selective absorption of light, thickness, light path length (according to the Beer-Lambert law), light sources [15], background colors [16], and human vision [17]. A material with specific selective absorption properties may appear different under varying light sources due to differences in the spectral power distribution. Additionally, metamerism can occur, where two light sources with different energy distributions can appear the same color (illuminant metamerism), or the same light can produce different color perceptions in different observers (observer metamerism). Background color also plays a crucial role; light reflected from a background can significantly alter the color of a transparent mineral. Fluorescence effects, such as those observed in pinkish-red spinel from Myanmar, can further influence color perception under daylight. Despite the use of color matching functions to predict color perception, the impact of surrounding factors is challenging to quantify precisely. This limitation has led to the development of color appearance models (CAMs). The CIECAM02 model, introduced by the Commission Internationale de l’Éclairage (CIE), was the first widely adopted model for describing perceptual color aspects [18–20]. The CAM16 model followed, building upon years of research. These models account for various perceptual factors when studying the color appearance of transparent minerals.

For precise color analysis of spinels, a modern color system such as the CIE1976 L^*^a^*^b^*^ uniform color space system is essential. This system has been widely used in mineralogical colorimetry [21–23]. Although the CIE1931 color space system enjoyed widespread popularity in past decades, the geometric distances it represents between colors fail to closely mirror human visual perception. In contrast, despite its own limitations, the CIE1976 system offers a more favorable alternative, as it aligns more closely with how humans perceive color differences, such as distortions in the a^*^-b^*^ diagram and uneven hue angles. While various alternative color systems have been proposed, the CIE1976 L^*^a^*^b^*^ system remains the standard due to its applicability and universality.

In this study, the colors of orange, red, purple, and blue spinel were analyzed under D65 and A light sources against nine Munsell neutral backgrounds. The color mechanisms of colored spinel and the effects of light sources and backgrounds on color perception were discussed. Color evaluation systems for colored spinel were established using Energy-Dispersive X-ray Fluorescence (ED-XRF), optical spectroscopy (UV-Vis-NIR), and spectrophotometry.

## Materials and methods

### Samples

In this research, 412 facet spinel crystals from Myanmar (Burma) were analyzed. There are 12 colored spinel crystals (from Spi-1 to Spi-12, each one weighs more than two carats) for color genesis study and chemical analysis. Besides, 100 purple, red, orange, and blue spinel crystals each for color testing (each one weighs more than one carat). All spinel crystals are in high transparency and clarity. The spinel samples were commercially obtained in 2021 from Fire Stone Jewelry Co., Ltd. in China. This study was conducted under the academic supervision and approval of Dean of the School of Gemmology, China University of Geosciences (Beijing). The research protocol was reviewed and approved by the supervisor, ensuring compliance with the laboratory safety regulations and academic ethics guidelines of the university. All experimental procedures and sample analyses were carried out in the laboratories of the School of Gemmology, with the use of facilities authorized by the supervisor.

### Optical absorption spectroscopy

Optical absorption spectra were measured in the 300–800 nm range using a UV-3600 UV-Vis-NIR spectrophotometer at room temperature and a 0.5 nm spectral resolution at a scan speed of 100 nm/min. The detector conversion wavelength is 850 nm and the grating conversion wavelength is 900 nm with S/R shift.

### ED-XRF analysis

The chemical composition of spinel was analyzed by Thermo Fisher Quant’X EDXRF. The conditions are as follow: 2 mm collimator; Low Za with 4 kV for 100 seconds; Low Zc with 12 kV and Al filter for 100 seconds; Mid Za with 16 kV and Pd thin filter for 100 seconds; Mid Zb with 20 kV Pd medium filter for 100 seconds.

### CIE1976 L*a*b* uniform color space system

The color system is composed of colorimetric coordinates a^*^, b^*^, and lightness L^*^. Thereinto, + a^*^ represents red while –a^*^ represents green, and +b^*^ represents yellow while –b^*^ represents blue. Chroma *C*_ab_^*^ and hue angle *h*_*ab*_^*°*^ should be calculated by a^*^ and b^*^:


$$C_{ab}^{*}={\left[{\left(a^{*}\right)}^{2}+{\left(b^{*}\right)}^{2}\right]}^{1/2}$$ (1)

$$h_{ab}^{\circ }=arctan\frac{b^{*}}{a^{*}}$$ (2)

To calculate the color difference of spinels, CIE DE2000 (Δ*E*_*00*_) formula which was recommended by CIE was applied. Compared with the CIE *LAB* (Δ*E*_ab_^*^) formula which lacks visual uniformity [24], Δ*E*_*00*_ calculates color difference more precisely in the green area, it’s an improved version of Δ*E*_ab_^*^. The formula is as follows:


$${\Delta E}_{00}={\left[{\left(\frac{{\Delta L}^{\prime }}{k_{L}S_{L}}\right)}^{2}+{\left(\frac{{\Delta C}^{\prime }}{k_{C}S_{C}}\right)}^{2}+{\left(\frac{{\Delta H}^{\prime }}{k_{H}S_{H}}\right)}^{2}+R_{T}{\left(\frac{{\Delta C}^{\prime }}{k_{C}S_{C}}\right)}^{2}\left(\frac{{\Delta H}^{\prime }}{k_{H}S_{H}}\right)\right]}^{1/2}$$ (3)

Δ*L*^*’*^, Δ*C*^*’,*^ and Δ*H*^*’*^ are lightness difference, chroma difference, and hue angle difference of a pair of color data, respectively. *R*_*T*_ is a function to reduce the interaction between chroma and hue in the blue area. *S*_*L*_, *S*_*C,*_ and *S*_*H*_ are functions to calibrate the absence of visual uniformity of the CIE *LAB* formula. *K*_*L*_, *K*_*C,*_ and *K*_*H*_ are correction parameters of the environment. There are two widely used combinations of (*K*_*L*_, *K*_*C*_, and *K*_*H*_): CIE DE2000 (1:1:1) [25] and CIE DE2000 (2:1:1) [26]. CIE DE2000 (1:1:1) was applied in this study because it has a better perceptibility when evaluating color differences.

### Colorimetric analysis

**Fig 1:**
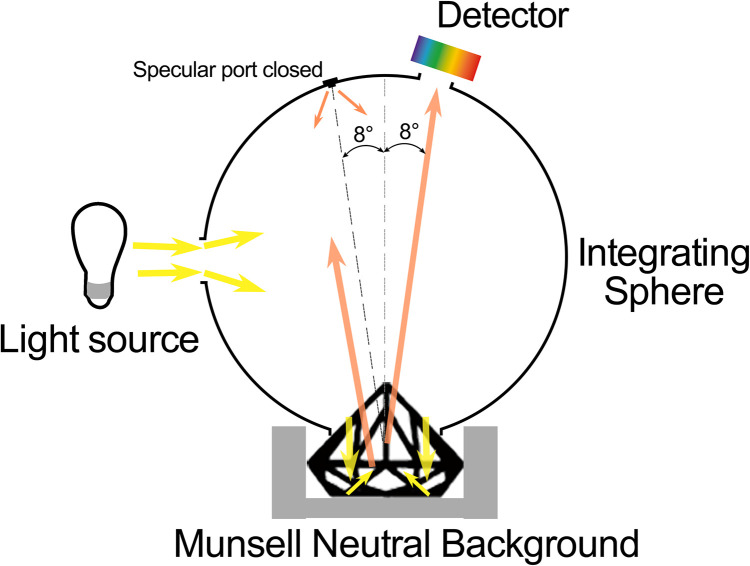
Color measurement experiment.

The spinel colors were acquired by an X-Rite Ci-7800 sphere benchtop spectrophotometer, measuring from 360 nm to 780 nm in a 5 nm wavelength interval in transmission mode with a 4 mm aperture. It measured for 4 seconds and used pulsed-xenon D65 calibrated lamp as illumination. The software X-Rite Color iQC was utilized to analyze and output color data. Munsell neutral value gray scales chart is a grayscale fan deck with values of 0.5/ to 9.5/, in quarter-step intervals. It is frequently used for instrumental calibration, imaging testing, or as reflection standards. Nine Munsell neutral value gray scales (glossy edition) with different gray scales or luminance factors (*Y*_b_) including N1 (*Y*_b_ = 1.210%), N2 (*Y*_b_ = 3.126%), N3 (*Y*_b_ = 6.555%), N4 (*Y*_b_ = 12.000%), N5 (*Y*_b_ = 19.770%), N6 (*Y*_b_ = 30.050%), N7 (*Y*_b_ = 43.060%), N8 (*Y*_b_ = 59.100%), and N9 (*Y*_b_ = 78.660%) were utilized as backgrounds in this experiment. A standard illumination box was adopted in the experiment. Different light source has different correlated color temperature (CCT) and relative spectral power distribution. Two standard light sources including A (CCT 2856K) and D65 (CCT 6504K) were used in this research. When measuring the colors of the spinel samples, each sample was encircled by various gray scale neutral backgrounds. As light penetrated through the table (top facet) of the spinel, the majority of it escaped from the pavilion of the stone and was then reflected back by the surrounding background. Subsequently, this reflected light was reabsorbed by the spinel and redirected into the integrating sphere, which allowed for our comprehensive color measurement and analysis (Fig 1).

## Results and discussions

### Chemical analysis

**Fig 2:**
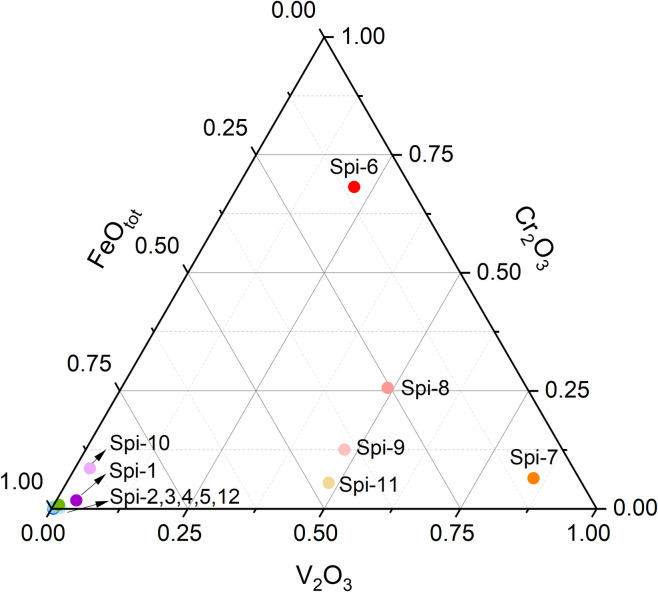
Normalized concentrations of FeO_tot_, Cr_2_O_3_, and V_2_O_3_ for the investigated spinel crystals.

Twelve chemically isotropic colored spinel crystals were analyzed using Energy-Dispersive X-ray Fluorescence (ED-XRF) (see Table 1, and S1 File). All the spinel samples are primarily composed of MgAl_2_O_4_, with the exception of one blue spinel containing a small amount of Zn (Spi-2). As an allochromatic mineral, spinel’s color cannot be solely attributed to its major elements [27–28]. Trace element analysis identified three main groups of spinel crystals: those containing Cr with minor Fe, those dominated by Fe, and those dominated by V. The key trace elements responsible for color in the investigated spinels include Fe (up to 3.30 wt% FeO_tot_), Cr (up to 1.56 wt% Cr_2_O_3_), V (up to 1.05 wt% V_2_O_3_), Co (up to 0.01 wt% Co_3_O_4_), and Mn (up to 0.09 wt% MnO), among others. According to the chemical composition plot (Fig 2), red spinel is primarily influenced by Cr, while orange color may be controlled by V. Purple, green, and blue hues in spinel are largely affected by Fe, though blue coloration may also be influenced by Co. Additionally, pink spinel may result from a combination of Fe, V, Cr, and other elements. The detailed color genesis of spinel will be further explored through UV-Vis-NIR analysis.

**Table 1:** EDXRF chemical compositions of investigated spinel crystals (wt%).

Sample	Color	ZnO	Ga_2_O_3_	Al_2_O_3_	MgO	TiO_2_	V_2_O_3_	Cr_2_O_3_	MnO	FeO_tot_	Co_3_O_4_	NiO	Total
Spi-1	Purple	0.1449%	0.0478%	70.8800%	27.1800%	0.0593%	0.0604%	0.0297%	0.0188%	1.5718%	0.0000%	0.0016%	99.9943%
Spi-2	Blue	4.2218%	0.0165%	66.6460%	27.6400%	0.0102%	0.0212%	0.0028%	0.0170%	1.4019%	0.0088%	0.0109%	99.9970%
Spi-3	Blue	0.5388%	0.0532%	70.2300%	25.8100%	0.0085%	0.0098%	0.0000%	0.0519%	3.2954%	0.0000%	0.0092%	100.0068%
Spi-4	Cyan	0.2811%	0.0393%	70.7370%	26.8100%	0.0000%	0.0083%	0.0000%	0.0907%	2.0294%	0.0000%	0.0000%	99.9957%
Spi-5	Green	0.1226%	0.0619%	70.8400%	27.4500%	0.0047%	0.0025%	0.0048%	0.0674%	1.4485%	0.0000%	0.0005%	100.0029%
Spi-6	Red	0.7558%	0.0981%	70.3400%	26.3800%	0.1120%	0.4892%	1.5622%	0.0153%	0.2373%	0.0000%	0.0095%	99.9994%
Spi-7	Orange	0.0965%	0.0857%	70.6100%	27.9000%	0.0614%	1.0514%	0.0799%	0.0029%	0.1032%	0.0000%	0.0013%	99.9923%
Spi-8	Pink	0.7912%	0.0543%	70.7100%	27.5600%	0.0256%	0.4098%	0.2153%	0.0056%	0.2142%	0.0000%	0.0123%	99.9983%
Spi-9	Pink	0.5028%	0.0462%	71.0700%	27.9400%	0.0210%	0.1952%	0.0516%	0.0034%	0.1646%	0.0000%	0.0082%	100.0029%
Spi-10	Light pink	2.2553%	0.0766%	70.2330%	26.1300%	0.0076%	0.0346%	0.1081%	0.0327%	1.1213%	0.0000%	0.0024%	100.0015%
Spi-11	Light pink	0.1973%	0.0490%	71.6400%	27.7800%	0.0113%	0.1511%	0.0173%	0.0037%	0.1458%	0.0000%	0.0016%	99.9971%
Spi-12	Colorless	0.7569%	0.0737%	71.5200%	27.3400%	0.0100%	0.0026%	0.0023%	0.0294%	0.2699%	0.0000%	0.0004%	100.0052%

### Optical absorption spectroscopy

**Fig 3:**
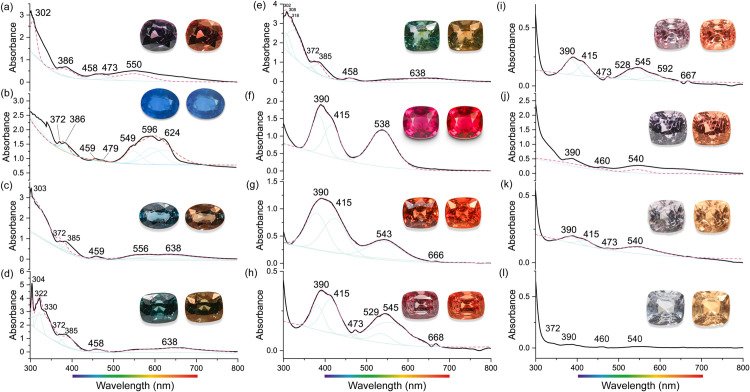
UV-Vis-NIR spectra of investigated spinel crystals. The pictures of spinel crystals illuminated by D65 (left) and A (right) standard lamp are displayed next to the spectrum.

The UV-Vis-NIR spectra of the 12 investigated spinel crystals (S2 Table) were measured in the range of 300–800 nm (Fig 3). The observed absorption peaks (Table 2) are consistent with previous studies [29–33].

**Table 2:** Optical absorption peaks (nm) of the investigated spinel crystals.

Lable	Spi-1	Spi-2	Spi-3	Spi-4	Spi-5	Spi-6	Spi-7	Spi-8	Spi-9	Spi-10	Spi-11	Spi-12	Assignment
*a*	–	372	372	372	372	–	–	–	–	–	–	372	s.f. ^5^E → ^3^E of ^T^Fe^2+^
*b*	386	386	385	385	385	390	390	390	390	390	390	390	s.f. ^5^E → ^3^T_2_, ^3^T_1_ of ^T^Fe^2+^
*c*	–	–	–	–	–	415	415	415	415	–	415	–	s.a. ^4^A_2g_ → ^4^T_1g_(F) of ^M^Cr^3+^
*d*	458	459	459	458	458	–	–	–	–	460	–	460	s.f. ^6^A_1g_ → ^4^A_1g_, ^4^E_g_ transition of ^M^Fe^3+^
	473	479	–	–	–	–	–	473	473	–	473	–	
*e*	–	–	–	–	–	538	543	545	545	540	540	540	s.a. ^4^A_2g_ → ^4^T_2g_(F) of ^M^Cr^3+^
*f*	550	549	556	–	–	–	–	–	–	–	–	–	s.a. ^3^T_1_(F) → ^3^T_2_(F) of ^M^V^3+^
*g*	–	596	–	–	–	–	–	–	–	–	–	–	s.f. ^5^E → ^3^T_1_ of ^T^Fe^2+^ s.a. ^4^A_2_(F) → ^4^T_1_(P) of ^T^Co^2+^
*h*	–	624	638	638	638	–	–	–	–	–	–	–	s.a. ^4^A_2_(F) → ^4^T_1_(P) of ^T^Co^2+^
*i*	–	–	–	–	–	–	666	668	667	–	–	–	^M^Fe^2+^ ↔ ^M^Fe^3 +^ IVCT ^T^Fe^2+^ ↔ ^M^Fe^3 +^ ECP

*s.f.* spin-forbidden, *s.a.* spin-allowed, *IVCT* intervalence charge transfer, *ECP* exchange coupled pair. The assignment of absorption bands to different transitions was made in agreement with previous studies [29–33].

Iron-bearing spinel crystals, which exhibit colors such as purple, blue, and green, show similar absorption features in their spectra. Peaks around ~372 nm, and ~385 nm (labeled *a* and *b*, respectively) are attributed to spin-forbidden transitions ^5^E → ^3^E of ^T^Fe^2^, and ^5^E → ^3^T_2_ with ^3^T_1_ of ^T^Fe^2+^, respectively. Sharp peaks at ~458 nm or ~473 nm (labeled *d*) could be assigned to spin-forbidden ^6^A_1g_ → ^4^A_1g_, ^4^E_g_ transition of isolated ^M^Fe^3+^ ions. The peak at 596 nm (labeled *g*) can be assigned to either spin-forbidden ^5^E → ^3^T_1_ of ^T^Fe^2+^ or spin-allowed ^4^A_2_(F) → ^4^T_1_(P) of ^T^Co^2+^, which may also generate peaks around ~624 nm or 638 nm (labeled *h*) simultaneously.

In chromium-bearing spinel crystals (typically red) and vanadium-bearing spinel crystals (typically orange), which contain only trace amounts of Fe, a peak around 390 nm is observed in their optical absorption spectra. Additionally, three of the investigated orange spinel crystals (Spi-7, 8, and 9) exhibit absorption peaks around ~667 nm (labeled *i*). This peak is assigned to intervalence charge transfer of ^M^Fe^2+^ ↔ ^M^Fe^3+^, as well as exchange coupled pair of ^T^Fe^2+^ ↔ ^M^Fe^3+^, in line with previous research [33]. The absorption peak at 415 nm (labeled *c*) is attributed to spin-allowed ^4^A_2g_ → ^4^T_1g_(F) of ^M^Cr^3+^, while the peak around ~540 nm (labeled *e*) is assigned to the spin-allowed ^4^A_2g_ → ^4^T_2g_(F) of ^M^Cr^3+^. Finally, the absorption peak at ~550 nm (labeled *f*) is attributed to spin-allowed ^3^T_1_(F) → ^3^T_2_(F) of ^M^V^3+^.

### Color analysis

Gem crystals can be categorized into isotropic and anisotropic types based on their crystallization structures [34]. Understanding crystallization is crucial when measuring crystal colors, as anisotropic crystals exhibit pleochroism, meaning they absorb light differently depending on the direction of observation. In contrast, isotropic crystals absorb light uniformly in all directions. Furthermore, gem crystals are typically cut and polished into facet stones to enhance their vivid colors, brilliance, and clarity. The facets of a gemstone refract and reflect light, creating a vibrant appearance that intensifies its color.

While there is research on calculating color from the table of anisotropic gemstones, such as chrysoberyl [35], measuring color from gemstone tables presents challenges due to the variability in cutting styles. This paper focuses on spinel, an isotropic crystal that does not exhibit pleochroism. To avoid uncertainties introduced by facets, we measured and observed the colors from the pavilion of faceted spinel crystals—a method long used in the evaluation of near-colorless diamonds [36].

Different light sources have distinct correlated color temperatures (CCT) and relative spectral power distributions (RSPD). Standard light sources include A (2856K), F12 (3000K), F11 (4000K), F2 (4230K), D50 (5003K), D65 (6504K), and D75 (7500K), among others. The A light source, an incandescent lamp commonly used in homes and stores, predominantly emits light in the yellow and red wavelengths. In contrast, D65 light sources simulate average daylight in the Northern Hemisphere [37] and are widely used in color evaluation across various industries. Since A and D65 are common in daily settings and bright fields are preferable for assessing gemstone colors, this study examines the color effects of A and D65 light sources on spinel crystals against a Munsell N9 neutral background (luminance factor = 0.78660, lightness = 91.08).

The colors of 400 investigated spinel crystals were measured using a spectrophotometer (see Table 3, and S3 Table). Illumination with a D65 light source revealed the following color metrics:

**Table 3:** Colors of spinel crystals under D65 and A standard light source.

Light source			Orange	Red	Purple	Blue
D65	Color parameters average	$\overset{―}{L^{*}}$	62.50	62.16	65.31	61.60
		$\overset{―}{a^{*}}$	13.72	23.10	9.37	−4.68
		$\overset{―}{b^{*}}$	15.15	1.33	−2.34	−0.75
		$\overset{―}{C^{*}}$	20.43	23.13	9.66	4.74
		$\overset{―}{h^{o}}$	47.84	3.31	345.97	9.07
	Color difference	Orange	–	–	–	–
		Red	16.70	–	–	–
		Purple	18.24	14.55	–	–
		Blue	24.34	27.86	14.62	–
A	Color parameters average	$\overset{―}{L^{*}}$	65.16	64.66	66.14	61.05
		$\overset{―}{a^{*}}$	19.30	25.39	9.87	−3.76
		$\overset{―}{b^{*}}$	17.22	4.87	−0.72	−2.42
		$\overset{―}{C^{*}}$	25.87	25.86	9.90	4.47
		$\overset{―}{h^{o}}$	41.74	10.86	355.82	32.80
	Color difference	Orange	–	–	–	–
		Red	13.78	–	–	–
		Purple	20.30	16.57	–	–
		Blue	30.58	30.27	14.65	–
Average color difference (D65-A)		7.09		5.11	1.85	2.34

Orange Spinel Crystals: Lightness ranged from 52.01 to 73.57, with an average of 62.50. Chroma ranged from 10.80 to 26.85, averaging 20.43. Hue angles varied from 31.07° to 63.10°, with an average of 47.84°.

Red Spinel Crystals: Lightness ranged from 50.50 to 75.69, with an average of 62.16. Chroma ranged from 13.58 to 30.55, averaging 23.13. Hue angles ranged from −10.78° (349.22°) to 18.89°, with an average of 3.31°.

Purple Spinel Crystals: Lightness ranged from 52.19 to 82.55, with an average of 65.31. Chroma ranged from 3.76 to 12.15, averaging 9.66. Hue angles ranged from −21.45° (338.55°) to −9.30° (350.70°), with an average of −14.03° (345.97°).

Blue Spinel Crystals: Lightness ranged from 51.27 to 74.36, with an average of 61.60. Chroma ranged from 2.90 to 6.14, averaging 4.74. Hue angles ranged from −1.64° (358.36°) to 19.84°, with an average of 9.07°.

Under A light source, the color metrics were as follows

Orange Spinel Crystals: Lightness ranged from 55.15 to 74.71, with an average of 65.16. Chroma ranged from 14.07 to 33.34, averaging 25.87. Hue angles ranged from 32.53° to 48.11°, with an average of 41.74°.

Red Spinel Crystals: Lightness ranged from 54.09 to 76.92, with an average of 64.66. Chroma ranged from 14.15 to 36.42, averaging 25.86. Hue angles ranged from −3.12° (356.88°) to 24.51°, with an average of 10.86°.

Purple Spinel Crystals: Lightness ranged from 53.04 to 82.84, with an average of 66.14. Chroma ranged from 4.33 to 12.61, averaging 9.90. Hue angles ranged from −13.56° (346.44°) to 1.71°, with an average of −4.18° (355.82°).

Blue Spinel Crystals: Lightness ranged from 50.65 to 74.03, with an average of 61.05. Chroma ranged from 2.41 to 5.18, averaging 4.47. Hue angles ranged from 19.82° to 41.23°, with an average of 32.80°.

Significant color differences between the four types of spinel crystals were observed when illuminated by D65 versus A light sources, corresponding to visible changes perceived by the naked eye. Notably, the color differences were significant for orange and red spinel crystals but less so for purple and blue spinel crystals.

**Fig 4:**
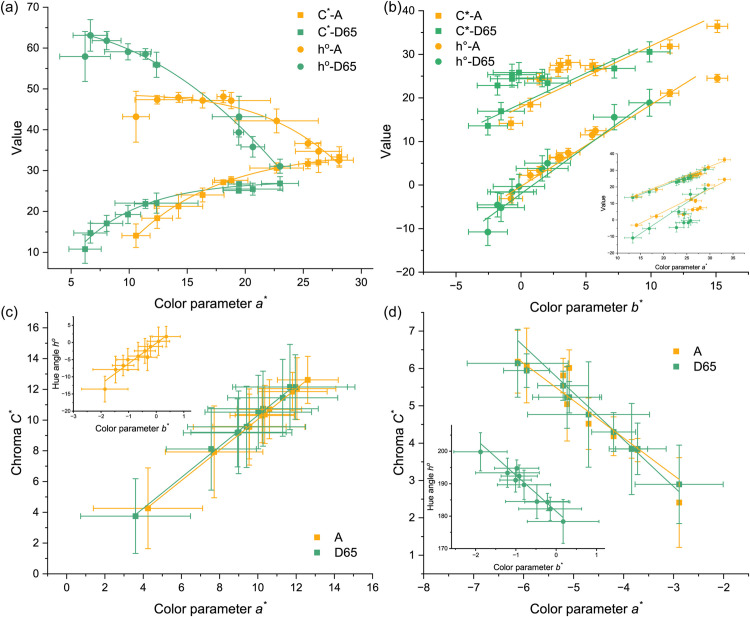
Correlations between color parameters (lightness L^*^, chroma C^*^, hue angle *h*^*o*^, color parameter a^*^ and b^*^) of orange (a), red (b), purple (c), and blue (d) spinel crystals under standard D65 and A lamps.

When illuminated by both D65 and A light sources, the chroma of orange spinel crystals showed a positive correlation with the color parameter a^*^, while the hue angle exhibited a negative correlation (Fig 4a). This suggests that the chroma and hue of orange spinel are influenced by the red tone under both daylight and incandescent lighting conditions.

For red spinel crystals, both chroma and hue angle positively correlated with the color parameters a^*^ and b^*^ (Fig 4b). This indicates that red and yellow tones significantly enhance the color of red spinel.

In the case of purple spinel, chroma exhibited a linear positive correlation with a^*^ under both D65 and A light sources, while the hue angle showed a positive correlation with -b^*^ (Fig 4c). This strongly suggests that chroma is significantly influenced by a^*^ in purple spinel, and its hue is more readily affected by the blue tone.

Blue spinel crystals displayed distinct trends in the relationship between color parameters. The chroma of blue spinel negatively correlated with -a^*^, and the hue negatively correlated with -b^*^ (Fig 4d). This indicates that the chroma of blue spinel is influenced by the green tone, while its hue is affected by the blue tone, which is consistent with expectations.

The light source is not the sole factor affecting the color of spinel crystals; background color also plays a significant role due to the high transparency of spinel. Therefore, considering the background color is crucial during color analysis and research. In this study, we analyzed and discussed the colorimetric characteristics of spinel under D65 and A light sources across nine Munsell neutral background colors.

The Munsell neutral value gray scales, also known as a Munsell gray scale or neutral color chart is a gray scale fan deck. Its lightness *L*_b_^*^ has a power function relationship with its luminance factor *Y*_*b*_, which is as follows:


$$L_{b}^{*}=116Y_{b}^{1/3}-16$$ (4)

**Fig 5:**
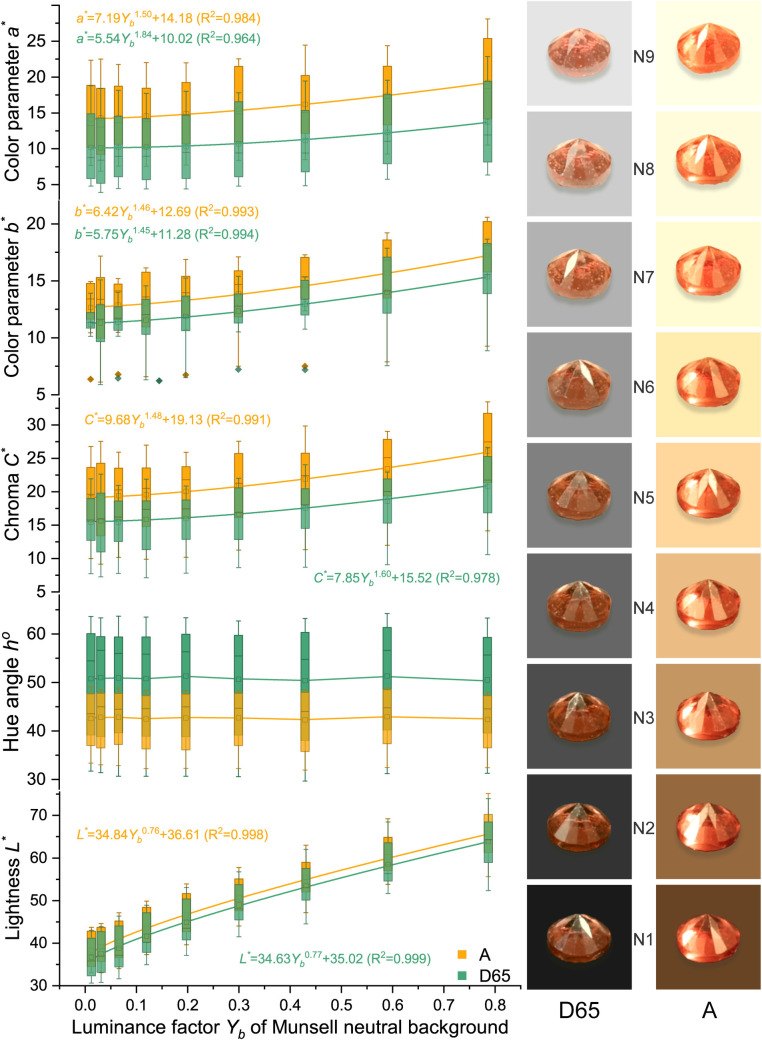
The color characteristic of orange spinel crystals on the nine Munsell neutral backgrounds under D65 and A standard light sources.

**Fig 6:**
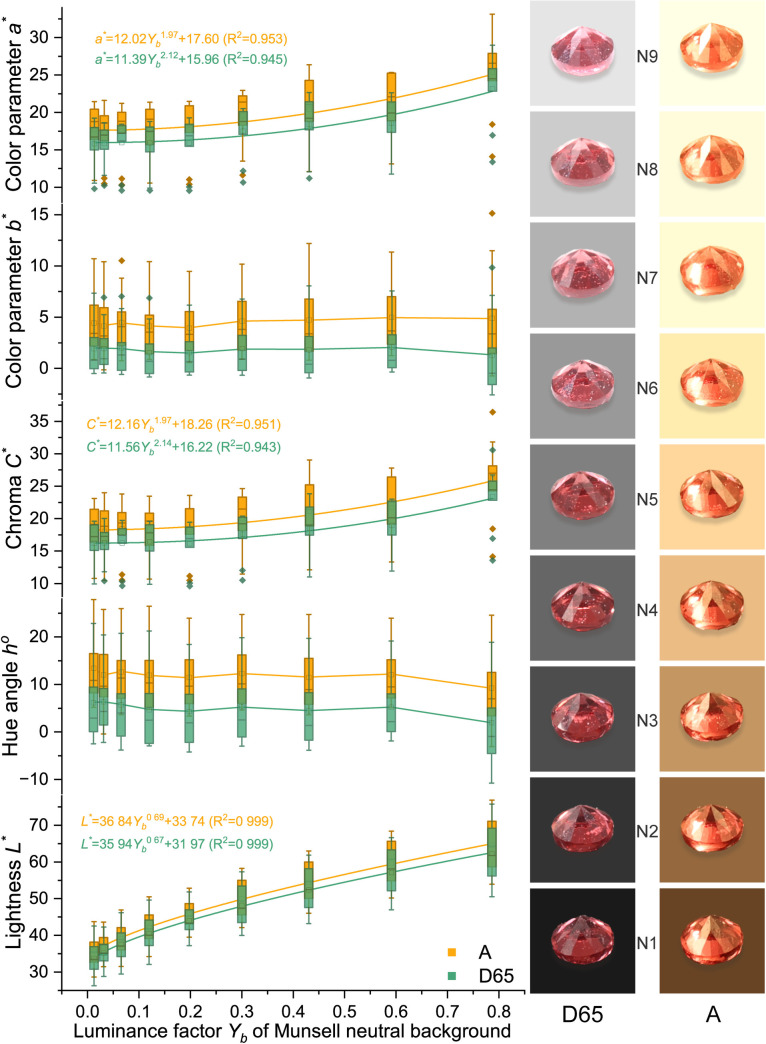
The color characteristic of red spinel crystals on the nine Munsell neutral backgrounds under D65 and A standard light sources.

**Fig 7:**
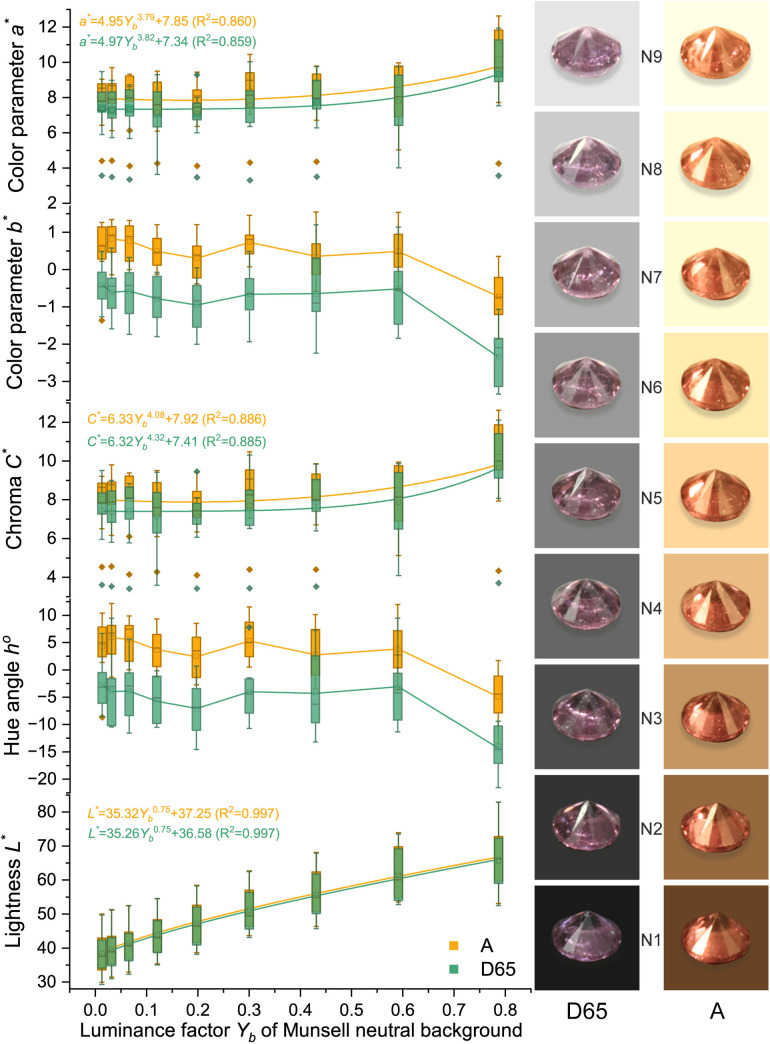
The color characteristic of purple spinel crystals on the nine Munsell neutral backgrounds under D65 and A standard light sources.

**Fig 8:**
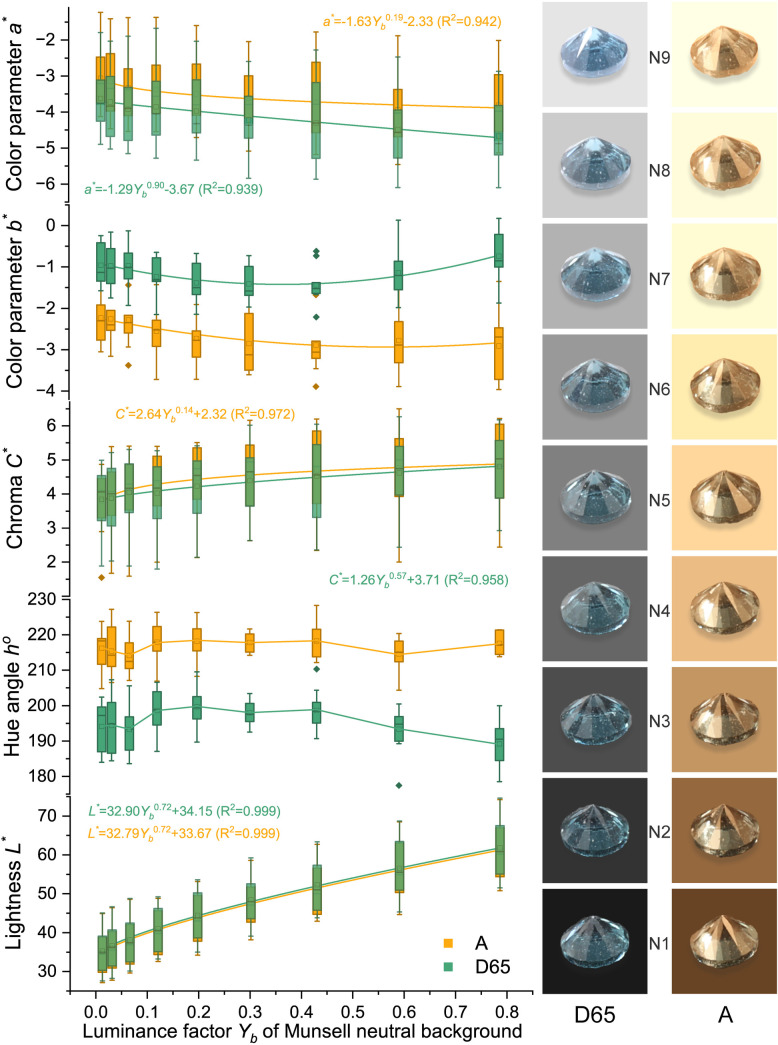
The color characteristic of blue spinel crystals on the nine Munsell neutral backgrounds under D65 and A standard light sources.

Due to the high grade of transparency of spinel, spinel colors should have a similar power function when the background color changes as Munsell gray scales. The colors were analyzed and presented in Fig 5 (orange), Fig 6 (red), Fig 7 (purple), and Fig 8 (blue). The lightness and chroma of all spinel colors increase significantly with the luminance factor of Munsell neutral background in power function relationship with different rate. However, the hue angle has no significant changes in the same process because gray could hardly affect other hue. The color parameters of some of the investigated spinel crystals show the similar trend with chroma because they have strong relation with each other for some specific color.

For yellow, red, purple, and blue spinel crystals, the power function relations between lightness and background luminance factor under two light sources are as follow:


$$L_{orange-D65}^{*}=34.63Y_{b}^{0.77}+35.02\;(R^{2}=0.999)$$ (5)

$$L_{orange-A}^{*}=34.84Y_{b}^{0.76}+36.61\;(R^{2}=0.998)$$ (6)

$$L_{red-D65}^{*}=35.94Y_{b}^{0.67}+31.97\;(R^{2}=0.999)$$ (7)

$$L_{red-A}^{*}=36.84Y_{b}^{0.69}+33.74\;(R^{2}=0.999)$$ (8)

$$L_{purple-D65}^{*}=35.26Y_{b}^{0.75}+36.58\;(R^{2}=0.997)$$ (9)

$$L_{purple-A}^{*}=35.32Y_{b}^{0.75}+37.25\;(R^{2}=0.997)$$ (10)

$$L_{blue-D65}^{*}=32.90Y_{b}^{0.72}+34.15\;(R^{2}=0.999)$$ (11)

$$L_{blue-A}^{*}=32.79Y_{b}^{0.72}+33.67\;(R^{2}=0.999)$$ (12)

There are similar relationships between chroma and background luminance factor:


$$C_{orange-D65}^{*}=7.85Y_{b}^{1.60}+15.52\;(R^{2}=0.978)$$ (13)

$$C_{orange-A}^{*}=9.68Y_{b}^{1.48}+19.13\;(R^{2}=0.991)$$ (14)

$$C_{red-D65}^{*}=11.56Y_{b}^{2.14}+16.22\;(R^{2}=0.943)$$ (15)

$$C_{red-A}^{*}=12.16Y_{b}^{1.97}+18.26\;(R^{2}=0.951)$$ (16)

$$C_{purple-D65}^{*}=6.32Y_{b}^{4.32}+7.41\;(R^{2}=0.885)$$ (17)

$$C_{purple-A}^{*}=6.33Y_{b}^{4.08}+7.92\;(R^{2}=0.886)$$ (18)

$$C_{blue-D65}^{*}=1.26Y_{b}^{0.57}+3.71\;(R^{2}=0.958)$$ (19)

$$C_{blue-A}^{*}=2.64Y_{b}^{0.14}+2.32\;(R^{2}=0.972)$$ (20)

The colors of spinel crystals could be easily predicted by the equations above by the luminance factor of the background under different light source.

### Color clustering

Clustering is an explorative data analysis technique used for investigating the underlying structure in the data. It described as the grouping of objects, where the objects share similar characteristics [38]. The classic K-means clustering algorithm finds cluster centroids that minimize the distance between data points and the nearest centroid [39]. K-means clustering analysis is effective and it has been applied in gemstone color research for the latest five years [40–41].

In this study, the K-means clustering method was utilized to categorize and classify the colors of four types of spinel crystals based on their lightness and chroma, using the Munsell color system. To ensure objectivity and universality, color clustering was performed on spinel crystals illuminated by the D65 standard light source against a Munsell N9 neutral background.

**Fig 9:**
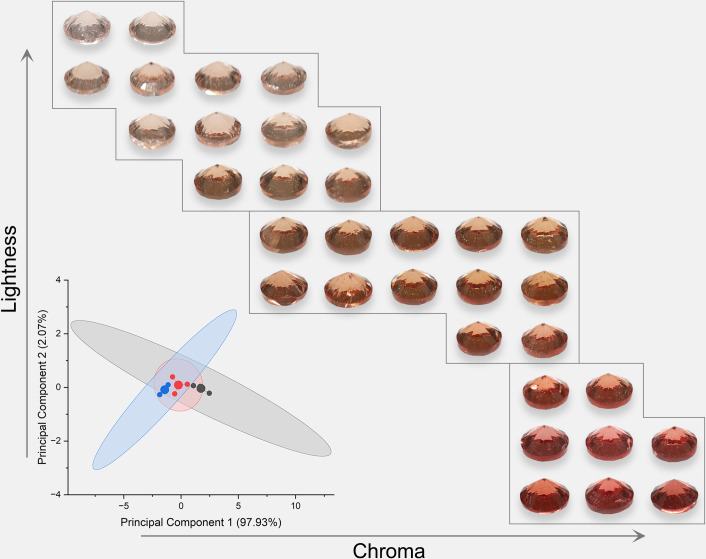
The color clustering of orange spinel crystals according to lightness and chroma.

**Fig 10:**
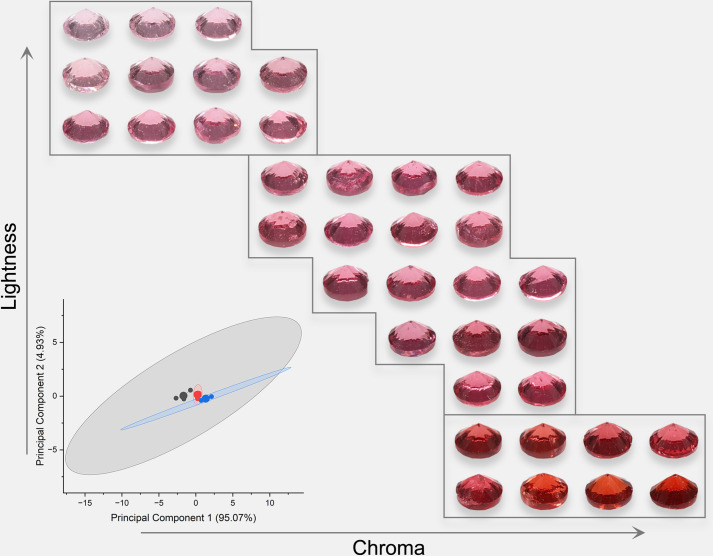
The color clustering of red spinel crystals according to lightness and chroma.

**Fig 11:**
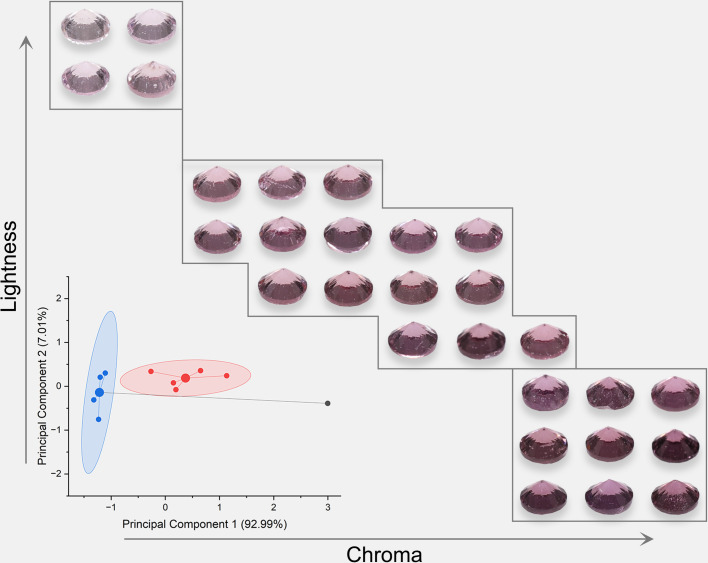
The color clustering of purple spinel crystals according to lightness and chroma.

**Fig 12:**
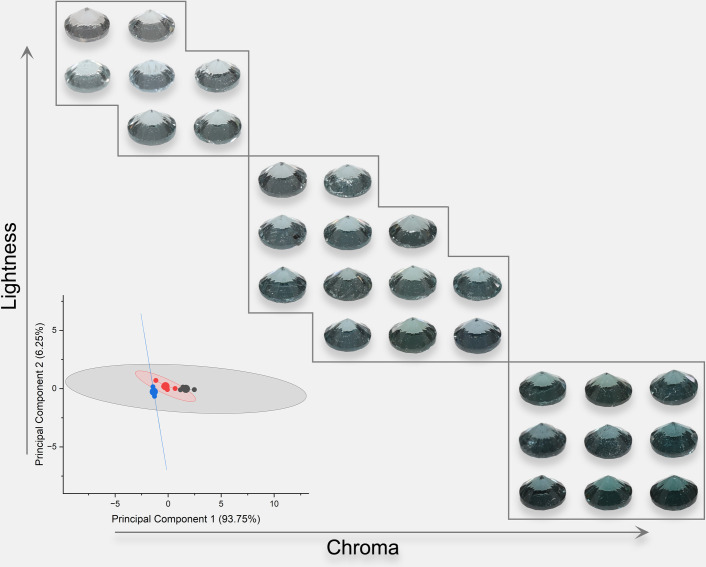
The color clustering of blue spinel crystals according to lightness and chroma.

The analysis was conducted using OriginPro software (version 2024b). The clustering results were notably improved when each type of spinel color was divided into three groups (Table 4). The color differences between these three groups for each type of spinel are sufficiently large to be perceptible to the human eye, surpassing the minimum color tolerance threshold [42]. For instance, the clustering analysis for orange spinel crystals yielded a P-value of 0.00139, indicating a significant result. This finding suggests that orange spinel colors can be effectively grouped into three distinct categories based on variations in lightness and chroma (Fig 9). Similarly, significant P-values of 0.01661, < 0.001, and 0.01135 were obtained for red (Fig 10), purple (Fig 11), and blue (Fig 12) spinel crystals, respectively. These results demonstrate the effectiveness of the clustering approach in distinguishing between different spinel colors and underscore its importance in developing a reliable color grading system for spinel.

**Table 4:** K-means clustering analysis of four types of spinel crystals.

Color	Group	Final cluster center (C^*^, L^*^)	Color difference			P
			Group-1	Group-2	Group-3	
Orange	1	(14.20, 70.88)	–	–	–	0.00139
	2	(22.08, 61.80)	12.02	–	–	
	3	(25.91, 55.06)	19.68	7.75	–	
Red	1	(17.79, 71.44)	–	–	–	0.01661
	2	(24.72, 61.58)	12.05	–	–	
	3	(27.28, 53.64)	20.17	8.34	–	
Purple	1	(3.76, 82.55)	–	–	–	<0.001
	2	(9.36, 69.02)	14.64	–	–	
	3	(11.57, 56.37)	27.32	12.84	–	
Blue	1	(3.53, 70.49)	–	–	–	0.01135
	2	(5.11, 61.41)	9.21	–	–	
	3	(5.57, 52.98)	17.63	8.44	–	

## Conclusions

Both the chroma and hue of orange spinel crystals are influenced by the red tone under both daylight and incandescent lighting conditions. In contrast, red and yellow tones significantly enhance the color of red spinel crystals. For purple spinel, chroma shows a strong correlation with the a^*^ parameter under both D65 and A light sources, while the hue is readily affected by blue tones. Blue spinel, on the other hand, has its chroma controlled by green tones and its hue influenced by blue tones.

The lightness and chroma of all spinel colors increase significantly with the luminance factor of the Munsell neutral background, following a power function relationship at different rates. However, the hue angle remains relatively unchanged during this process, as gray backgrounds have minimal impact on hue. The colors of spinel crystals can be effectively predicted using calculated equations based on the luminance factor of the background under various light sources. Additionally, employing the K-means clustering method to separate each spinel color into three groups proves effective in classifying the colors.

## Supporting information

S1 File Chemical compositions of the spinels. (DOCX)

S2 Table UV-Vis spectra of the spinels. (XLSX)

S3 Table Color data of the spinels. (XLSX)

S4 Inclusivity-in-global-research-questionnaire. (DOCX)

## References

[1] Cuthill IC, Allen WL, Arbuckle K, Caspers B, Chaplin G, Hauber ME, et al. The biology of color. Science. 2017;357(6350):eaan0221. doi: 10.1126/science.aan0221 28774901

[2] Conway BR, Malik-Moraleda S, Gibson E. Color appearance and the end of Hering’s Opponent-Colors Theory. Trends Cogn Sci. 2023;27(9):791–804. doi: 10.1016/j.tics.2023.06.003 37394292 PMC10527909

[3] Halenius U, Bosi F. Color of Mn-bearing gahnite: A first example of electronic transitions in heterovalent exchange coupled IVMn2+-VIMn3+ pairs in minerals. American Mineralogist. 2014;99(2–3):261–6. doi: 10.2138/am.2014.4670

[4] Zhao Q, Yan Z, Chen C, Chen J. Spinels: Controlled Preparation, Oxygen Reduction/Evolution Reaction Application, and Beyond. Chem Rev. 2017;117(15):10121–211. doi: 10.1021/acs.chemrev.7b00051 28745484

[5] Wang M, Wang M, Qi Y, Xue Y, Shi G. Chemical Composition and Spectral Characteristics of Different Colored Spinel Varieties from Myanmar. Minerals. 2024;14(11):1124. doi: 10.3390/min14111124

[6] Pardieu V. Hunting for “Jedi” Spinels in Mogok. G&G. 2014;50(1):46–57. doi: 10.5741/gems.50.1.46

[7] Wu J, Sun X, Ma H, Ning P, Tang N, Ding T, et al. Purple-Violet Gem Spinel from Tanzania and Myanmar: Inclusion, Spectroscopy, Chemistry, and Color. Minerals. 2023;13(2):226. doi: 10.3390/min13020226

[8] Chauvire B, Rondeau B, Fritsch E, Ressigeac P, Devidal J-L. Blue Spinel from the Luc Yen District of Vietnam. G&G. 2015;51(1):2–17. doi: 10.5741/gems.51.1.2

[9] Belley P, Palke A. Purple Gem Spinel from Vietnam and Afghanistan: Comparison of Trace Element Chemistry, Cause of Color, and Inclusions. G&G. 2021;57(3):228–38. doi: 10.5741/gems.57.3.228

[10] Chankhantha C, Amphon R, Rehman HU, Shen AH. Characterisation of Pink-to-Red Spinel from Four Important Localities. Journ of Gemm. 2020;37(4):393–403. doi: 10.15506/jog.2020.37.4.393

[11] Brik MG, Suchocki A, Kamińska A. Lattice parameters and stability of the spinel compounds in relation to the ionic radii and electronegativities of constituting chemical elements. Inorg Chem. 2014;53(10):5088–99. doi: 10.1021/ic500200a 24784795

[12] Nassau K. The fifteen causes of color: The physics and chemistry of color. Color Research & Application. 1987;12(1):4–26. doi: 10.1002/col.5080120105

[13] Halenius U, Andreozzi GB, Skogby H. Structural relaxation around Cr3+ and the red-green color change in the spinel (sensu stricto)-magnesiochromite (MgAl2O4-MgCr2O4) and gahnite-zincochromite (ZnAl2O4-ZnCr2O4) solid-solution series. American Mineralogist. 2010;95(4):456–62. doi: 10.2138/am.2010.3388

[14] Andreozzi GB, D’Ippolito V, Skogby H, Hålenius U, Bosi F. Color mechanisms in spinel: a multi-analytical investigation of natural crystals with a wide range of coloration. Phys Chem Minerals. 2018;46(4):343–60. doi: 10.1007/s00269-018-1007-5

[15] Liu Y, Shigley J, Fritsch E, Hemphill S. The “alexandrite effect” in gemstones. Color Research & Application. 1994;19(3):186–91. doi: 10.1002/col.5080190306

[16] Tang J, Guo Y, Xu C. Metameric effects on peridot by changing background color. J Opt Soc Am A Opt Image Sci Vis. 2019;36(12):2030–9. doi: 10.1364/JOSAA.36.002030 31873376

[17] Li C, Li Z, Wang Z, Xu Y, Luo MR, Cui G, et al. Comprehensive color solutions: CAM16, CAT16, and CAM16‐UCS. Color Research & Application. 2017;42(6):703–18. doi: 10.1002/col.22131

[18] CIE Publication 159:2004. A colour appearance model for colour management systems: CIECAM02. Vienna: CIE Central Bureau. 2004. https://cie.co.at/publications/colour-appearance-model-colour-management-systems-ciecam02

[19] Moroney N, Fairchild MD, Hunt RWG, Li CJ, Luo MR, Newman T. The CIECAM02 colour appearance models. The Tenth Color Imaging Conference: Color Science and Engineering Systems, Technologies, Applications, CIC 2002, November 12, 2002, Scottsdale, Arizona, USA. 2002: 23–27.

[20] Li CJ, Luo MR, Hunt RWG, Moroney N, Fairchild MD, Newman T. The performance of CIECAM02. The Tenth Color Imaging Conference: Color Science and Engineering Systems, Technologies, Applications, CIC 2002, November 12, 2002, Scottsdale, Arizona, USA, 2002: 28–32.

[21] Cheng R, Guo Y. Study on the effect of heat treatment on amethyst color and the cause of coloration. Sci Rep. 2020;10(1):14927. doi: 10.1038/s41598-020-71786-1 32913246 PMC7483767

[22] Wang X, Guo Y. The impact of trace metal cations and absorbed water on colour transition of turquoise. R Soc Open Sci. 2021;8(2):201110. doi: 10.1098/rsos.201110 33972843 PMC8074670

[23] Zhang S, Guo Y. Measurement of Gem Colour Using a Computer Vision System: A Case Study with Jadeite-Jade. Minerals. 2021;11(8):791. doi: 10.3390/min11080791

[24] Commission Internationale de I’Éclairage. Proceedings of the 8th Session of the CIE, Cambridge. 1931.

[25] Commission Internationale de I’Éclairage. CIE technical report: Improvement to industrial colour-difference evaluation. CIE Publication. 2001. https://cie.co.at/publications/improvement-industrial-colour-difference-evaluation

[26] Perez MDM, Ghinea R, Herrera LJ, Ionescu AM, Pomares H, Pulgar R, et al. Dental ceramics: a CIEDE2000 acceptability thresholds for lightness, chroma and hue differences. J Dent. 2011;39 Suppl 3:e37-44. doi: 10.1016/j.jdent.2011.09.007 21986320

[27] Andreozzi GB, Princivalle F, Skogby H, Della Giusta A. Cation ordering and structural variations with temperature in MgAl2O4spinel: An X-ray single-crystal study. American Mineralogist. 2000;85(9):1164–71. doi: 10.2138/am-2000-8-907

[28] Andreozzi GB, Lucchesi S, Skogby H, della Giusta A. Compositional dependence of cation distribution in some synthetic (Mg,Zn)(Al,Fe3+)2O4 spinels. ejm. 2001;13(2):391–402. doi: 10.1127/0935-1221/01/0013-0391

[29] Hålenius U, Skogby H, Andreozzi GB. Influence of cation distribution on the optical absorption spectra of Fe 3+ -bearing spinel s.s. -hercynite crystals: evidence for electron transitions in VI Fe 2+ - VI Fe 3+ clusters. Physics and Chemistry of Minerals. 2002;29(5):319–30. doi: 10.1007/s00269-002-0240-z

[30] Taran MN, Koch-Müller M, Langer K. Electronic absorption spectroscopy of natural (Fe2+, Fe3+)-bearing spinels of spinel s.s.-hercynite and gahnite-hercynite solid solutions at different temperatures and high-pressures. Phys Chem Minerals. 2005;32(3):175–88. doi: 10.1007/s00269-005-0461-z

[31] D’Ippolito V, Andreozzi GB, Bosi F, Hålenius U, Mantovani L, Bersani D, et al. Crystallographic and spectroscopic characterization of a natural Zn-rich spinel approaching the endmember gahnite (ZnAl2O4) composition. Mineral mag. 2013;77(7):2941–53. doi: 10.1180/minmag.2013.077.7.05

[32] Fregola RA, Skogby H, Bosi F, D’Ippolito V, Andreozzi GB, Halenius U. Optical absorption spectroscopy study of the causes for color variations in natural Fe-bearing gahnite: Insights from iron valency and site distribution data. American Mineralogist. 2014;99(11–12):2187–95. doi: 10.2138/am-2014-4962

[33] D’Ippolito V, Andreozzi GB, Hålenius U, Skogby H, Hametner K, Günther D. Color mechanisms in spinel: cobalt and iron interplay for the blue color. Phys Chem Minerals. 2015;42(6):431–9. doi: 10.1007/s00269-015-0734-0

[34] Kožíšek Z. Crystallization in small droplets: Competition between homogeneous and heterogeneous nucleation. Journal of Crystal Growth. 2019;522:53–60. doi: 10.1016/j.jcrysgro.2019.06.007

[35] Sun Z, Palke AC, Muyal J, McMurtry R. How to facet gem-quality chrysoberyl: Clues from the relationship between color and pleochroism, with spectroscopic analysis and colorimetric parameters. American Mineralogist. 2017;102(8):1747–58. doi: 10.2138/am-2017-6011

[36] King JM, Geurts RH, Gilbertson AM, Shigley JE. Color Grading “D-to-Z” Diamonds at the GIA Laboratory. Gems & Gemology. 2008;44(4):296–321. doi: 10.5741/gems.44.4.296

[37] Lam Y, Xin JH. Evaluation of the quality of different D65 simulators for visual assessment. Color Research & Application. 2002;27(4):243–51. doi: 10.1002/col.10061

[38] Govender P, Sivakumar V. Application of k-means and hierarchical clustering techniques for analysis of air pollution: A review (1980–2019). Atmospheric Pollution Research. 2020;11(1):40–56. doi: 10.1016/j.apr.2019.09.009

[39] Coates A, Ng AY. Learning feature representations with k-means. In: Montavon G, Orr GB, Müller KR, editors. Neural Networks: Tricks of the Trade - Second Edition. Springer. 2012.

[40] Liu Z, Guo Y. The Effect of Munsell Neutral Value Scale on the Color of Yellow Jadeite and Comparison between AP and K-Means Clustering Color Grading Schemes. Crystals. 2022;12(2):241. doi: 10.3390/cryst12020241

[41] Lv H, Guo Y. Genesis of the Body Color of Brazilian Gem-Quality Yellow-Green Opal. Crystals. 2023;13(2):316. doi: 10.3390/cryst13020316

[42] Liu HX, Wu B, Liu Y, Huang M, Xu YF. A Discussion on Printing Color Difference Tolerance by CIEDE2000 Color Difference Formula. AMM. 2012;262:96–9. doi: 10.4028/www.scientific.net/amm.262.96

