# Supplementary material for: Colorimetry characteristics and color clustering of natural gem-quality spinel from Myanmar (Burma)
Source: PLoS One. 2025 Aug 14;20(8):e0312054. doi: 10.1371/journal.pone.0312054 (PMC12352828; doi:10.1371/journal.pone.0312054)
Supplement: S1 File — (DOCX) [file pone.0312054.s001.docx]

**
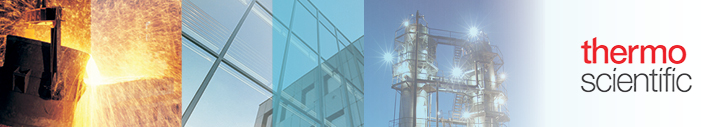
**

**EDXRF Analysis Report**

Thermo Fisher Scientific Inc.

**Sample List:** 2024/02/21 10:59 **Analyzed:** Wed Feb 21 12:01:19 2024

**Analysis Technique:** Fundamental Parameters (Theoretical) **Last Calibrated:** Thu Jun 13 21:09:17 2019

**Method File:** C:\...\Desktop\METHODS\2023\10\Spinel-SpinOn-June19-Final(2)NEW.mth **Software version:** 10.7.0.20352

**Comments:**

**Conditions**

**Mid Zb**

Voltage 20 kV Current Auto

Livetime 100 seconds Counts Limit 0

Filter Pd Medium Atmosphere Air

Maximum Energy 40 keV Count Rate Medium

Warmup time 2 seconds

**Low Za**

Voltage 4 kV Current Auto

Livetime 100 seconds Counts Limit 0

Filter No Filter Atmosphere Vacuum

Maximum Energy 40 keV Count Rate Medium

Warmup time 2 seconds

**Low Zc**

Voltage 12 kV Current Auto

Livetime 100 seconds Counts Limit 0

Filter Al Atmosphere Air

Maximum Energy 40 keV Count Rate Medium

Warmup time 2 seconds

**Mid Za**

Voltage 16 kV Current Auto

Livetime 100 seconds Counts Limit 0

Filter Pd Thin Atmosphere Air

Maximum Energy 40 keV Count Rate Medium

Warmup time 2 seconds

**Results**

**Element Concentration Peak(cps/mA) Background(cps/mA)**

**1 Spinel purple by Joseph**

ZnO 0.1449 % 69 -2

Ga2O3 0.0478 % 14 0

Al2O3 70.88 % 1756 27

MgO 27.18 % 292 10

TiO2 0.0593 % 7 1

V2O5 0.0604 % 10 1

Cr2O3 0.02969 % 9 3

MnO 0.01879 % 8 2

Fe2O3 1.5718 % 341 -15

Co3O4 0 % 0 31

NiO 0.00160 % 1 0

**2 Spinel blue by Joseph**

ZnO 4.2218 % 2146 -62

Ga2O3 0.0165 % 5 12

Al2O3 66.646 % 1768 -27

MgO 27.64 % 308 19

TiO2 0.01017 % 1 1

V2O5 0.02115 % 4 1

Cr2O3 0.00282 % 1 2

MnO 0.01695 % 9 2

Fe2O3 1.4019 % 358 -12

Co3O4 0.0088 % 3 37

NiO 0.01089 % 6 2

**3 Spinel blue by Joseph**

ZnO 0.5388 % 244 -8

Ga2O3 0.0532 % 14 1

Al2O3 70.23 % 1802 16

MgO 25.81 % 281 12

TiO2 0.0085 % 1 2

V2O5 0.00979 % 2 1

Cr2O3 0 % 0 3

MnO 0.05192 % 24 -2

Fe2O3 3.2954 % 740 -30

Co3O4 0 % 0 67

NiO 0.00921 % 4 0

**4 Spinel cyan by Joseph**

ZnO 0.2811 % 164 -5

Ga2O3 0.0393 % 14 1

Al2O3 70.737 % 2187 27

MgO 26.81 % 358 16

TiO2 [0.00019] % 0 2

V2O5 0.00827 % 2 0

Cr2O3 [0.00023] % 0 2

MnO 0.09067 % 51 -4

Fe2O3 2.0294 % 551 -22

Co3O4 0 % 0 48

NiO 0 % 0 1

**5 Spinel green by Joseph**

ZnO 0.1226 % 66 -2

Ga2O3 0.0619 % 20 0

Al2O3 70.84 % 1961 20

MgO 27.45 % 331 13

TiO2 0.00465 % 1 1

V2O5 0.00249 % 0 1

Cr2O3 0.00480 % 2 2

MnO 0.06738 % 34 -3

Fe2O3 1.4485 % 353 -15

Co3O4 0 % 0 32

NiO 0.00053 % 0 0

**6 Spinel red by Joseph**

ZnO 0.7558 % 264 -10

Ga2O3 0.0981 % 21 1

Al2O3 70.34 % 1292 55

MgO 26.38 % 208 20

TiO2 0.1120 % 9 0

V2O5 0.4892 % 59 0

Cr2O3 1.5622 % 332 -2

MnO 0.0153 % 5 47

Fe2O3 0.2373 % 36 -3

Co3O4 0 % 0 3

NiO 0.0095 % 3 0

**7 Spinel orange by Joseph**

ZnO 0.0965 % 45 -1

Ga2O3 0.0857 % 24 -0

Al2O3 70.61 % 1620 9

MgO 27.90 % 281 8

TiO2 0.0614 % 6 -0

V2O5 1.0514 % 158 -3

Cr2O3 0.0799 % 21 22

MnO 0.00292 % 1 4

Fe2O3 0.1032 % 20 -1

Co3O4 0 % 0 2

NiO 0.00125 % 1 0

**8 Spinel pink by Joseph**

ZnO 0.7912 % 396 -13

Ga2O3 0.0543 % 16 2

Al2O3 70.71 % 1737 36

MgO 27.56 % 294 12

TiO2 0.0256 % 3 0

V2O5 0.4098 % 67 -1

Cr2O3 0.2153 % 62 8

MnO 0.00560 % 3 10

Fe2O3 0.2142 % 46 -2

Co3O4 0 % 0 5

NiO 0.01233 % 6 0

**9 Spinel pink by Joseph**

ZnO 0.5028 % 294 -10

Ga2O3 0.0462 % 16 2

Al2O3 71.07 % 1995 23

MgO 27.94 % 344 12

TiO2 0.0210 % 3 0

V2O5 0.1952 % 36 -1

Cr2O3 0.0516 % 17 11

MnO 0.00336 % 2 4

Fe2O3 0.1646 % 41 -2

Co3O4 0 % 0 4

NiO 0.00818 % 5 0

**10 Spinel light pink by Joseph**

ZnO 2.2553 % 1292 -42

Ga2O3 0.0766 % 26 5

Al2O3 70.233 % 2083 28

MgO 26.13 % 328 16

TiO2 0.00756 % 1 1

V2O5 0.0346 % 7 1

Cr2O3 0.1081 % 38 1

MnO 0.03265 % 18 5

Fe2O3 1.1213 % 303 -12

Co3O4 0 % 0 28

NiO 0.00237 % 1 1

**11 Spinel light pink by Joseph**

ZnO 0.1973 % 123 -4

Ga2O3 0.0490 % 18 0

Al2O3 71.640 % 2136 39

MgO 27.78 % 364 15

TiO2 0.01125 % 1 0

V2O5 0.1511 % 30 -0

Cr2O3 0.01726 % 6 5

MnO 0.00374 % 2 4

Fe2O3 0.1458 % 38 -1

Co3O4 0 % 0 3

NiO 0.00161 % 1 2

**12 Spinel colorless by Joseph**

ZnO 0.7569 % 410 -13

Ga2O3 0.0737 % 24 1

Al2O3 71.52 % 1873 21

MgO 27.34 % 311 13

TiO2 0.01003 % 1 0

V2O5 0.0026 % 0 5

Cr2O3 0.00226 % 1 3

MnO 0.02941 % 14 0

Fe2O3 0.2699 % 63 -3

Co3O4 0 % 0 6

NiO 0.00042 % 0 1
